# Retraction: Enhanced antimicrobial treatment by a clay-based drug nanocarrier conjugated to a guanidine-rich cell penetrating peptide

**DOI:** 10.1039/d4ra90148g

**Published:** 2024-12-16

**Authors:** Mohammad Reza Khodabakhshi, Mohammad Hadi Baghersad

**Affiliations:** a Applied Biotechnology Research Center, Baqiyatallah University of Medical Sciences Tehran Iran

## Abstract

Retraction of ‘Enhanced antimicrobial treatment by a clay-based drug nanocarrier conjugated to a guanidine-rich cell penetrating peptide’ by Mohammad Reza Khodabakhshi *et al.*, *RSC Adv.*, 2021, **11**, 38961–38976, https://doi.org/10.1039/D1RA07821F.

The Royal Society of Chemistry hereby wholly retracts this *RSC Advances* article due to concerns with the reliability of the data.

A section of Fig. 4b has been duplicated in Fig. 4e, but they represent different halloysite nanotubes (HNTs).

Panels in Fig. 7 labelled ‘*S. aureus*/Individual VCM’, ‘VCM/Individual VCM’, ‘merge/Individual VCM’ and ‘*E. coli*/VCM-nanocargo’ overlap with Fig. 12 in another publication by different authors (W. Zhang, R. Taheri-Ledari, Z. Hajizadeh, E. Zolfaghari, M. R. Ahghari, A. Maleki, M. R. Hamblin and Y. Tian, Enhanced activity of vancomycin by encapsulation in hybrid magnetic nanoparticles conjugated to a cell-penetrating peptide, *Nanoscale*, 2020, **12**, 3855–3870).

The authors claim they outsourced the confocal microscopy to an external laboratory and received the incorrect raw data. However, the author's response did not satisfactorily address the concerns.

Given the significance of the concerns about the validity of the data, the findings presented in this paper are no longer reliable.

Mohammad Hadi Baghersad disagrees with the retraction and Mohammad Reza Khodabakhshi has not responded.

Laura Fisher, Executive Editor, *RSC Advances*

15th November 2024

